# Antioxidant Activity and Neuroprotective Activity of Stilbenoids in Rat Primary Cortex Neurons via the PI3K/Akt Signalling Pathway

**DOI:** 10.3390/molecules23092328

**Published:** 2018-09-12

**Authors:** Haichao Wen, Zheng Fu, Yangji Wei, Xiaoxu Zhang, Liyan Ma, Liwei Gu, Jingming Li

**Affiliations:** 1 Center for Viticulture and Enology, College of Food Science and Nutritional Engineering, China Agricultural University, Beijing 100083, China; wenhc@cau.edu.cn (H.W.); zheng.fu2@mail.mcgill.ca (Z.F.); weiyangji@sinochem.com (Y.W.); 2 Department of Biochemistry, The Rosalind and Morris Goodman Cancer Centre, McGill University, Montreal, QC H3G 1Y6, Canada; 3 Sinochem Group Co., Ltd., Beijing 100031, China; 4 College of Food Engineering and Biotechnology, Tianjin University of Science & Technology, Tianjin 300457, China; zxxjoypeace@tust.edu.cn; 5 College of Food Science and Nutritional Engineering, China Agricultural University, Beijing 100083, China; lyma1203@cau.edu.cn; 6 Food Science and Human Nutrition Department, Institute of Food and Agricultural Sciences, University of Florida, Gainesville, FL 32611, USA; lgu@ufl.edu

**Keywords:** Alzheimer’s disease, β-amyloid, rat primary cortex neurons, stilbenoids, secondary metabolites, biological activity

## Abstract

Antioxidant activity and neuroprotective activity of three stilbenoids, namely, *trans*-4-hydroxystilbene (THS), *trans*-3,5,4′-trihydroxy-stilbene (resveratrol, RES), and *trans*-3′,4′,3,5-tetrahydroxy-stilbene (piceatannol, PIC), against β-amyloid (Aβ)-induced neurotoxicity in rat primary cortex neurons were evaluated. THS, RES, and PIC significantly scavenged DPPH• and •OH radicals. All three stilbenoids were able to inhibit Aβ neurotoxicity by decreasing intracellular reactive oxygen species (ROS) via the PI3K/Akt signalling pathway. Specifically, stilbenoids significantly promoted Akt phosphorylation; suppressed Bcl-2/Bax expression; and inhibited caspase-9, caspase-3, and PARP cleavage. Molecular docking between stilbenoids with Akt indicated that stilbenoids could form hydrogen bond interactions with the COOH-terminal region of Akt. Additionally, the neuroprotective activity of stilbenoids correlated with the number and position of hydroxyl groups. The lack of meta-dihydroxyl groups on THS did not affect its neuroprotective activity in comparison with RES, whereas the ortho-dihydroxyl moiety on PIC significantly enhanced neuroprotective activity. These results provide new insights into the correlation between the biological activity and chemical structure of stilbenoids.

## 1. Introduction

Alzheimer’s disease, the most common form of dementia worldwide, is a neurodegenerative disorder characterized by the loss of neurons in the brain and cognitive decline. The typical neuropathological hallmarks of Alzheimer’s disease are neurofibrillary tangles consisting of hyperphosphorylated tau and senile plaques deposited on β-amyloid (Aβ) peptides [1]. In the “amyloid cascade hypothesis” of Alzheimer’s disease pathogenesis, neurotoxic Aβ accumulation in the brain has been considered an important cause of Alzheimer’s disease development [2,3].

Aβ peptides are formed by proteolytic cleavage of the amyloid precursor protein (APP) by β-secretase and γ-secretase [4]. Many studies have confirmed that Aβ cytotoxicity is accompanied by oxidative stress [5,6,7]. The balance between the production and removal of reactive oxygen species (ROS) is paramount to cell survival. An imbalance toward the pro-oxidative state is often referred to as oxidative stress. The ROS overproduction caused by oxidative stress and Aβ result in direct oxidative damage to cellular DNA, lipids, and proteins, followed by cellular structural damage, cellular function disorder, and cell apoptosis [5]. In addition, ROS are capable of exerting toxicity on neurological processes through their interaction with neuronal signalling pathways. Aβ and ROS accumulation induces neurotoxicity by interacting with protein kinase and lipid kinase signalling cascades [8,9]. 

PI3K/Akt is a set of vital kinases that plays a pivotal role in the regulation of apoptosis. The inactivation of PI3K/Akt is one of the signalling pathways through which Aβ and ROS exert their neurotoxicity. Inactivated Akt regulates various pro-apoptotic mediators such as NF-κB [10], mTOR [11]. When unstimulated, Akt exists mainly in an unphosphorylated state. Unphosphorylated Akt may target the Bcl-2 family protein and induce mitochondrial dysfunction [12]. Mitochondrial dysfunction is an important event that leads to the stimulation of multiple downstream caspases that ultimately induce the apoptotic process [13]. Based on the notion that oxidative stress responses can be an attractive target, many natural bioactive compounds with antioxidant activity are considered to be potential therapeutics for Alzheimer’s disease [14]. Moreover, therapeutic efforts against Alzheimer’s disease using these compounds have focused on reducing and inhibiting Aβ-induced oxidative stress and neuron apoptosis. 

**Figure 1:**
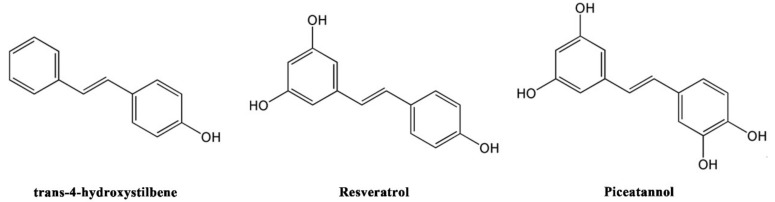
The chemical structure of three stilbenoinds.

Stilbenes consist of a series of derivatives with a basic structure of diphenylethylene (Figure 1), which are secondary metabolites in Vitis species and wine. Stilbenoids are hydroxylated derivatives of stilbene and display biological activities like antioxidant activity [15,16]. A previous study showed that monthly and weekly intake of wine was significantly associated with a lower risk of dementia and suggested that certain components, especially stilbenes in wine, may play an important role [17]. Rivière et al. indicated that 20 stilbenes, including resveratrol (RES) and piceatannol (PIC), inhibited Aβ peptide aggregation in vitro [18]. Feng et al. showed that RES inhibited Aβ-induced damage in PC12 cells by reducing oxidative stress via the SIRT1–ROCK1 signalling pathway [19]. Our previous study confirmed that two stilbenoids (PIC and pterostilbene) activated PI3K/Akt/Bad signalling pathway, and further suppressed Aβ-induced ROS generation and apoptotic activity in PC12 cells [20]. These studies indicated that stilbenoids possess neuroprotective activity. Moreover, numerous studies have shown that the antioxidative and antiproliferative activity of stilbenoids are related to their chemical structure [15,21,22]. 

To extend our knowledge of the neuroprotective effects of stilbenoids with different hydroxyl number and position, the protective activities of *trans*-4-hydroxystilbene (THS), RES, and PIC against Aβ_25-35_-induced neurotoxicity in rat primary cortex neurons were investigated and compared, and their underlying molecular mechanisms and signalling pathways were evaluated. We aimed to provide a basis for dietary intervention in adjunctive Alzheimer’s disease therapy and to clarify the relationship between the neuroprotective activity and chemical structure of stilbenoids.

## 2. Results

### 2.1. Free Radical Scavenging Activity of Stilbenoids on DPPH• and •OH

**Figure 2:**
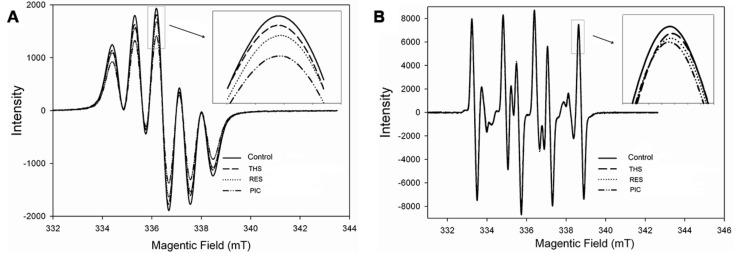
Electron paramagnetic resonance (EPR) spectra obtained from the reaction of stilbenoids with reactive oxygen species (ROS) (**A**) DPPH free radical and (**B**) OH free radical. The free radical scavenging activities were compared according to the decrease in peak intensity. THS—*trans*-4-hydroxystilbene; RES—resveratrol; PIC—piceatannol.

DPPH• and •OH were examined by electron paramagnetic resonance (EPR) spectroscopy to detect the presence of unpaired electrons. All three stilbenoids significantly decreased the concentrations of DPPH• and •OH free radicals (Figure 2). The inhibition of DPPH• free radicals of THS, RES, and PIC was 6.25%, 13.07%, and 26.82%, respectively. The peak intensity of •OH free radicals for stilbenoids was lower than that for the control (215.29, 296.85, and 438.55 A.U., arbitrary units). In addition, the scavenging activity of DPPH• and •OH followed the sequence PIC > RES > THS, which indicates that the antioxidant capacity of stilbenoid increased with the number of hydroxyl substituents to a certain extent. This result is consistent with the findings of Murias et al. [21], who showed that hydroxyl groups enhanced the antioxidant and anti-proliferative activities of resveratrol analogues.

### 2.2. Effects of Stilbenoids on Intracellular ROS in Aβ_25-35_-Induced Neurons 

**Figure 3:**
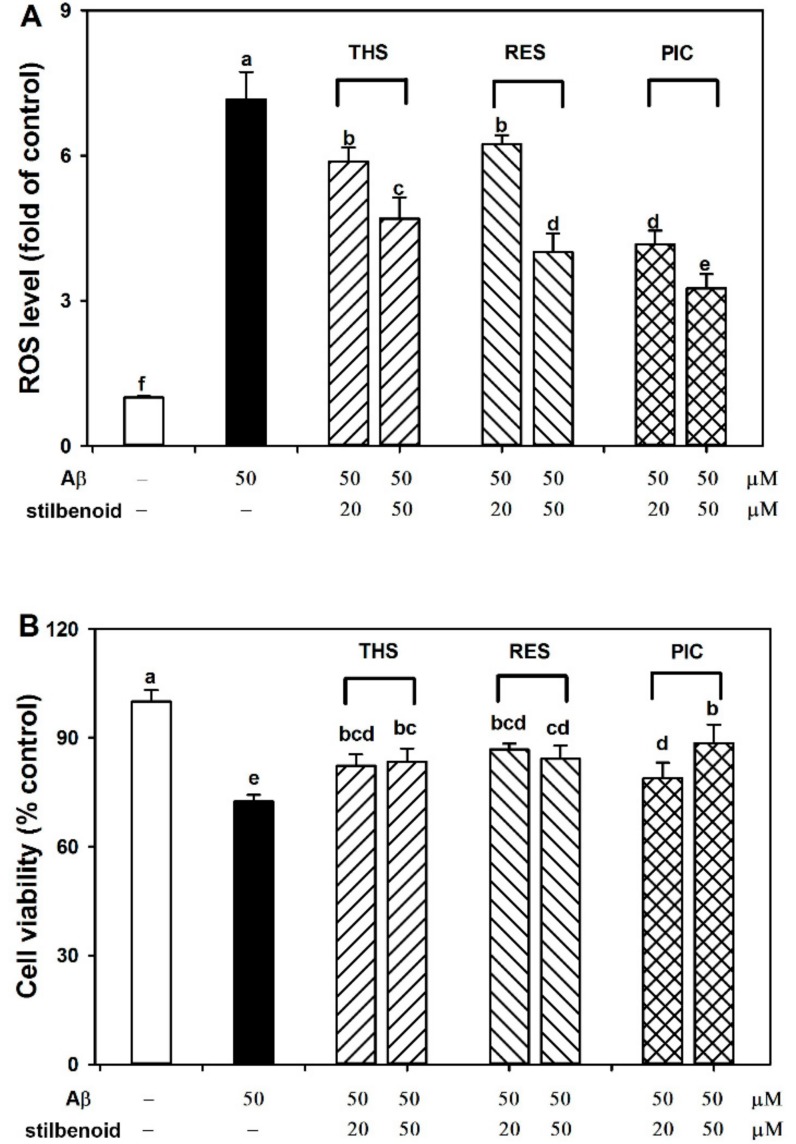
Effects of *trans*-4-hydroxystilbene (THS), resveratrol (RES), and piceatannol (PIC) on (**A**) intracellular ROS and (**B**) cell viability in Aβ_25-35_-induced rat primary cerebral cortex neurons. Each bar represents the mean ± SD of three independent experiments. Different letters in each figure indicate significant differences by Duncan’s multiple range test at *p* < 0.05.

Aβ cytotoxicity is accompanied by ROS accumulation and oxidative stress [5,6]. Overproduction of ROS causes oxidative injury to cells and act as mediators to regulate the signalling pathway [5,9]. Intracellular ROS is an important index for evaluating neuroprotective activity. Therefore, we detected intracellular ROS in Aβ_25-35_-induced neurons by 2′,7′-Dichlorofluorescin diacetate (DCFH-DA) probe. Figure 3A shows that Aβ_25-35_ significantly (*p* < 0.01) increased the amount of intracellular ROS (716%) relative to that in the control without Aβ_25-35_ treatment. All three stilbenoids at 20 and 50 μM caused a significant and dose-dependent decrease in ROS level (588%, 470% for THS; 624%, 401% for RES; 417%, 326% for PIC) compared with the Aβ_25-35_ treatment group (*p* < 0.01), suggesting that stilbenoids may help relieve oxidative stress and exert potential neuroprotective activity. In addition, the activity followed an order of PIC > RES > THS. The variation in the ROS level of neurons (Figure 3A) correlated closely with that of the free radical level (Figure 2). 

### 2.3. Effects of Stilbenoids on Cell Viability in Aβ_25-35_-Induced Neurons 

Neuron loss and apoptosis in the brain is a key characteristic of Alzheimer’s disease development, which is also an important result of Aβ-induced neurotoxicity [23]. To investigate the neuroprotective activity, primary neurons were treated with stilbenoids and Aβ_25-35_ and measured by 3-[4,5-Dimethylthiazol-2-yl]-2,5-diphenyl tetrazolium bromide (MTT) assay. As shown in Figure 3B, Aβ_25-35_ significantly (*p* < 0.05) reduced the cell viability (72%) compared with that of the control without Aβ_25-35_ treatment. The PIC treatment group (20, 50 μM) exhibited significantly increased cell viability (79%, 89%) compared with that of the Aβ_25-35_ treatment group (*p* < 0.05). Treatment with RES and THS (20, 50 μM) increased cell viability to similar extents (87%, 84% for RES; 82%, 83% for THS), although to a lesser extent than PIC. The observation is similar to previous results indicating that PIC was more prominent than RES in increasing cell viability against 4-hydroxynonenal, hydrogen peroxide, and peroxynitrite [24,25]. To confirm the neuroprotective activity, we next investigated the potential signalling pathways.

### 2.4. Effects of Stilbenoids on Akt Phosphorylation in Aβ_25-35_-Induced Neurons 

**Figure 4:**
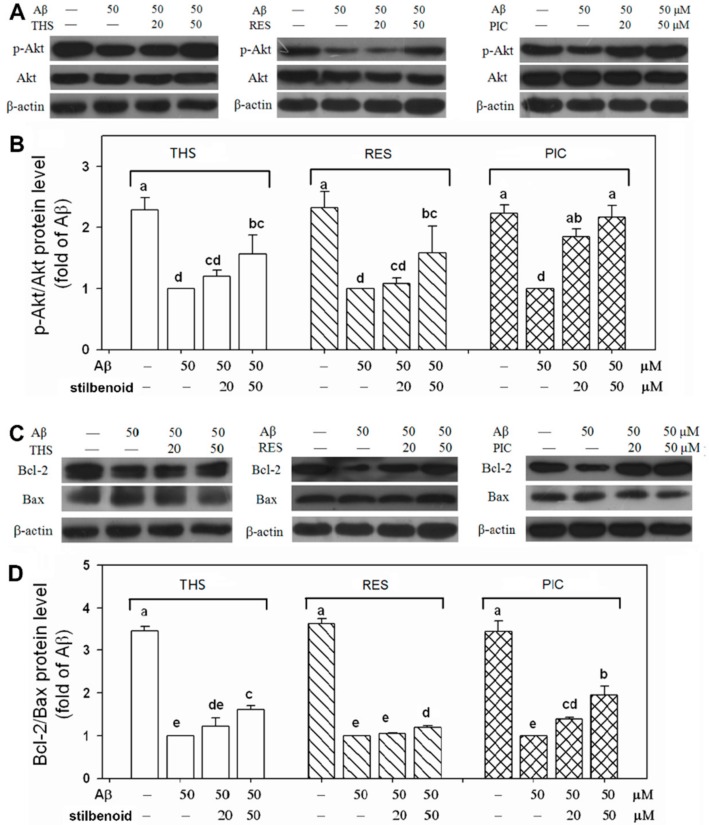
Effects of *trans*-4-hydroxystilbene (THS), resveratrol (RES), and piceatannol (PIC) on (**A**) Akt phosphorylation and (**C**) Bcl-2/Bax expression in Aβ_25-35_-induced rat primary cerebral cortex neurons. The relative protein levels of (**B**) p-Akt and (**D**) Bcl-2/Bax were calculated. Each bar represents the mean ± SD of three independent experiments. Different letters in each figure indicate significant differences by Duncan’s multiple range test at *p* < 0.01.

Akt, the most important factor in the PI3K/Akt signalling pathway, regulates numerous downstream factors to adjust cell apoptosis [26]. Akt phosphorylation at Ser-473 can activate the pathway to inhibit cell apoptosis and promote cell survival. We previously found that PIC and pterostilbene possess anti-apoptotic activity against Aβ-induced apoptosis in PC12 cells via the PI3K/Akt/Bad signalling pathway [20]. Therefore, we tested the influence of the three stilbenoids on activation of the PI3K/Akt signalling pathway by Western blotting. As shown in Figure 4A,B, Aβ_25-35_ decreased the level of p-Akt compared with the level measured for the control group without Aβ_25-35_ treatment (*p* < 0.01). PIC treatment (20, 50 μM) resulted in a promotion by 185%, 216% in Akt phosphorylation relative to that of the group with only Aβ_25-35_ treatment (*p* < 0.01). Furthermore, only THS and RES at 50 μM significantly (*p* < 0.01) increased the p-Akt level (157% for THS; 158% for RES), although the level was much lower than that observed for PIC. The results suggest that THS, RES, and PIC activated the PI3K/Akt signalling pathway to relieve Aβ damage and that the activity was related to their chemical structure.

### 2.5. Effects of Stilbenoids on Bcl-2 Family Proteins in Aβ_25-35_-Induced Neurons 

To further investigate the downstream signalling pathway, Bcl-2 family proteins in the mitochondria-mediated signalling pathway were examined. Bcl-2 family proteins are divided into anti-apoptotic proteins (Bcl-2, Bcl-xL, etc.) and pro-apoptotic proteins (Bax, Bad, Bak, etc.) [27]. Bcl-2/Bax is an important set of proteins related to apoptosis on mitochondria. As shown in Figure 4C,D, Aβ_25-35_ treatment significantly suppressed Bcl-2/Bax expression (*p* < 0.01) and showed pro-apoptotic activity. PIC (20, 50 μM) markedly (*p* < 0.01) reversed the change in a dose-dependent manner (139%, 194%). Moreover, THS showed passable neuroprotective activity, in which THS increased (*p* < 0.01), to a lesser extent than PIC, the Bcl-2/Bax level (122%, 161%). On the other hand, only RES at 50 μM significantly increased the level of Bcl-2/Bax (119%) compared with the level measured for the Aβ_25-35_-stimulated control group (*p* < 0.01), and the activity was much lower than that measured for THS and PIC. The results showed that PIC activated more Bcl-2 family proteins to anti-apoptosis than THS or RES.

### 2.6. Effects of Stilbenoids on Multiple Caspases in Aβ_25-35_-Induced Neurons 

**Figure 5:**
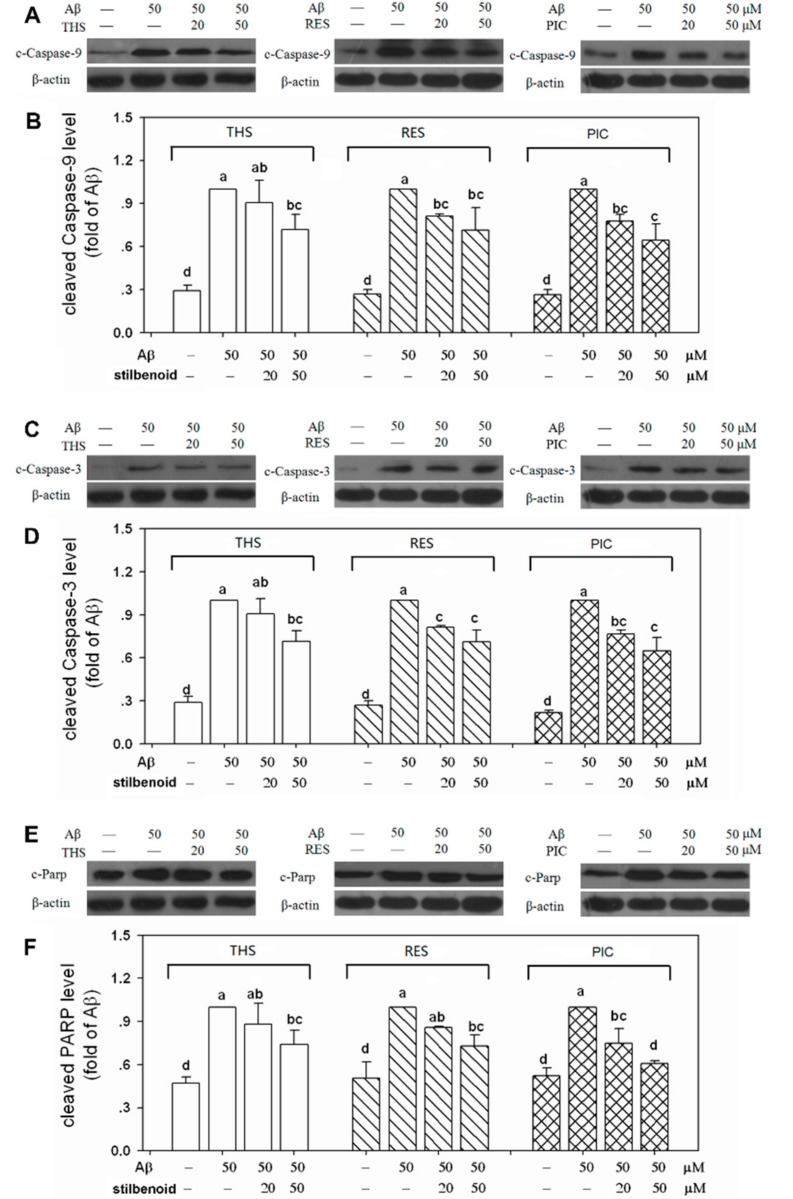
Effects of *trans*-4-hydroxystilbene (THS), resveratrol (RES), and piceatannol (PIC) on (**A**,**C**,**E**) multiple caspase expression in Aβ_25-35_-induced rat primary cortex neurons. Western blotting was used to analyse the cleavage of caspase-9, caspase-3, and PARP. The relative protein levels were calculated and are presented in (**B**,**D**,**F**). Each bar represents the mean ± SD of three independent experiments. Different letters in each figure indicate significant differences by Duncan’s multiple range test at *p* < 0.01.

When the expression of Bcl-2/Bax was suppressed, mitochondrial permeability increased and cytochrome c was released, which subsequently led to the cleavage and activation of multiple caspase cascades downstream to induce cell apoptosis [28]. As a result of the increased level of Bcl-2/Bax by THS, RES, and PIC treatments (Figure 4C,D), we further examined whether these three stilbenoids could inhibit the activation of multiple caspases. As illustrated in Figure 5, the levels of cleaved caspase-9, caspase-3, and cleaved PARP were significantly elevated (*p* < 0.01) in Aβ_25-35_-induced neurons. THS at 50 μM suppressed the activation of caspase-9 (72%), caspase-3 (72%), and PARP (74%). Moreover, RES at 20 and 50 μM showed somewhat better neuroprotective activity and reduced the levels of cleaved-caspase-9 (81%, 71%), cleaved-caspase-3 (82%, 72%), and cleaved-PARP (86%, 73%). In particular, PIC at concentrations of 20 and 50 μM significantly suppressed the cleavage of caspase-9 (78%, 64%), caspase-3 (77%, 65%), and PARP (75%, 61%). In addition, the level of cleaved-PARP when treated with PIC at 50 μM was much lower than that treated with THS and RES, suggesting that PIC exhibits more prominent neuroprotective activity. 

### 2.7. Molecular Docking of Stilbenoids with Akt Protein

**Figure 6:**
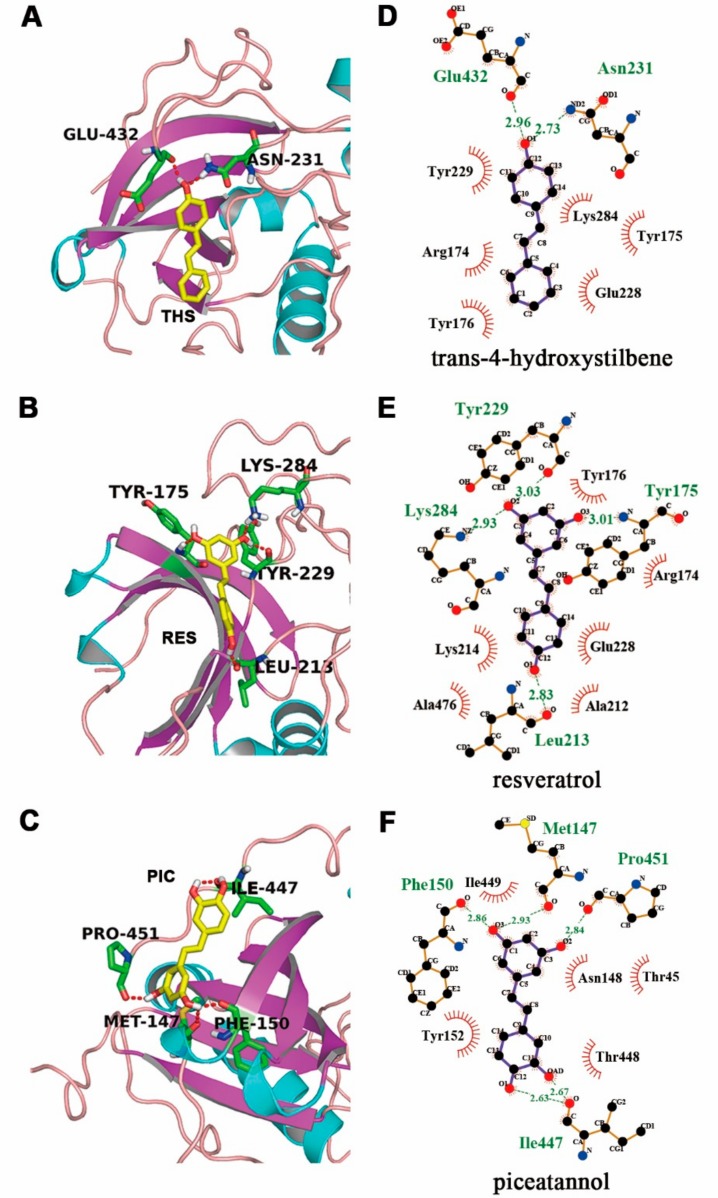
Molecular docking conformation of Akt protein binding with (**A**) *trans*-4-hydroxystilbene (THS), (**B**) resveratrol (RES), and (**C**) piceatannol (PIC). (**D**–**F**) Two-dimensional plots of the hydrophobic interactions between ligands and Akt protein. The atoms are color-coded as follows: carbon, black; nitrogen, blue; oxygen, red; and sulphur, yellow. The ligand’s bonds are shown in purple, and those of the protein residues are in orange. Hydrogen bonds between protein and ligand are represented by green dotted lines, lengths in angstroms; residues involved in nonbonded contacts with the ligand are represented by the arcs, with corresponding arcs on the relevant atoms of the ligand.

COOH-terminal is a crucial region for Akt induction and maintenance of its kinase activity [29,30]. Carboxy-terminal modulator protein (CTMP) is an important Akt-interacting protein binding to the COOH-terminal of Akt to inhibit Akt Phosphorylation on Ser473, resulting in a decrease in Akt activity [29,31]. To investigate the interaction, molecular docking was performed by Autodock 4 to uncover the combination mechanism between the stilbenoids ligand and the Akt receptor. We selected best-docking conformation based on the binding energy from the largest clusters among 100 runs. Table 1 shows that the best docking conformations of RES and PIC combined with the binding site of COOH-terminal yielded a binding energy of −6.69 and −6.73 kcal/mol and the inhibition constant (*K_i_*) value was 12.54 and 11.75 μM lower than THS. Van der Waals force (VDW), hydrogen bonding, and desolvation energy determined the intermolecular energy, and the energies of PIC was lower than RES and THS. The cartoon representations of Akt protein with three stilbenoids were shown by PyMOL to study the interaction (Figure 6A–C).

Ligand interaction plots showed different hydrogen bonds and hydrophobic interaction of three stilbenoids with Akt protein in Figure 6D–F using LigPlot+ analysis. In the optimal docked position, Akt protein formed two hydrogen bonds with THS, involving residues Asn231 and Glu432. Resveratrol formed four hydrogen bonds on Leu213, Lys284, Tyr229, and Tyr175. The residues of Akt protein such as Arg174, Tyr175, Tyr176, Ala212, Glu228, Tyr229, Lys284, Ala476, and Leu213 provided a major hydrophobic interface. Meanwhile, PIC formed five hydrogen bonds on Met147, Phe150, Ile447, and Pro451, which were hydrophobic amino acid residues formed a hydrophobic interface. In addition, Ile447 formed two hydrogen bonds (2.63 and 2.67 Å), of which the distance is more close. Besides, the binding energy increased by potentiating the π–π interactions between the ligand ring and aromatic residues of Phe150, Tyr152, Tyr175, Tyr176, and Tyr229. Regarding the binding energies and hydrogen bonds, the PIC appears to have the tightest binding with Akt, followed by RES and THS.

## 3. Discussion

Senile plaques deposited on Aβ peptides are one of the major histopathological hallmarks of Alzheimer’s disease. Aβ-induced neurotoxicity has been regarded as a key factor in Alzheimer’s disease onset [32,33]. Numerous studies have shown that Aβ accumulation in the brain results in oxidative stress in neurons, which leads to an increase in intracellular ROS. These result in activation of apoptosis-related signalling pathways and structural damage of neurons, and eventually causes neuron death or loss of function [6,34]. Therefore, natural bioactive compounds with strong antioxidant capabilities, such as EGCG, curcumin, and resveratrol, may possess potential neuroprotective activity and therapeutic effects against Alzheimer’s disease. 

### 3.1. Stilbenoids Suppress Aβ_25-35_-Induced Neurotoxicity by Reducing ROS via PI3k/Akt Signalling Pathway in Neurons

Among the three stilbenoids tested, RES [19,35,36] and PIC [20,24,25] had been proven with neuroprotective activity via various signalling pathways. No report regarding the neuroprotective property of THS against Aβ has been found to date. In this study, we observed that all three stilbenoids exhibit antioxidant ability, making them capable of scavenging the DPPH and OH free radicals (Figure 2). In addition, THS, RES, and PIC efficiently suppressed the accumulation of intracellular ROS (Figure 3A) and increased cell viability (Figure 3B) in Aβ_25-35_-treated primary cortex neurons. This neuroprotective effect appears to correlate with the PI3K/Akt signalling pathway. Specifically, THS, RES, and PIC promoted Akt phosphorylation (Figure 4A,B), promoted Bcl-2/Bax expression (Figure 4C,D), and inhibited multiple caspase cleavage (Figure 5). The results suggest that THS, RES, and PIC inhibit Aβ-induced neurotoxicity by decreasing intracellular ROS via the PI3K/Akt signalling pathway in primary neurons.

ROS plays an important role in the process of cell apoptosis. The antioxidant and free radicals scavenging ability of the three stilbenoids help to relieve oxidative stress and to prevent ROS from causing direct oxidative injury to lipids, proteins, and DNA, following by cell apoptosis. Moreover, THS, RES, and PIC are capable of exerting neuroprotective effects through their interaction with neuronal signalling pathways. In the present study, all three stilbenoids activated the PI3K/Akt pathway, promoting Akt phosphorylation on Ser473. Molecular docking results showed that all three stilbenoids are involved in forming hydrogen bond interactions with COOH-terminal region including the hydrophobic motif (HM) and COOH-terminal serine phosphorylation site of Akt. CTMP has been identified as an Akt inhibitor that binds to its COOH-terminal region [30]. CTMP prevents Akt activation at the plasma membrane in response to various stimulus [37]. There are many downstream targets for phosphorylated Akt. Liu et al. [35] indicated that RES suppressed high glucose-induced neurotoxicity via the PI3K/Akt/FoxO3a pathway. Xian et al. [38] showed that isorhynchophylline relieved Aβ neurotoxicity by promoting phosphorylation of GSK-3β, a downstream target of the PI3K/Akt pathway. Our results suggest that the stilbenoids targeted Bax and Bcl-2 near mitochondria to inhibit mitochondrial dysfunction (Figure 4c,d). This result is consistent with the finding of Zeng et al. [12], indicating that phosphorylated Akt promotes Bad phosphorylation, inhibits unphosphorylated Bad interacting with Bcl-xl on mitochondria, and subsequently relieves mitochondrial dysfunction. Stilbenoids prompted Bcl-2/Bax expression, which would inhibit cytochrome c release from mitochondrial, subsequently leading to the inactivation of multiple caspase cascades [28,39]. Therefore, combined with the above-mentioned results pertaining to caspases, these results provide solid evidence that THS, RES, and PIC inhibit Aβ-induced neurotoxicity via the PI3K/Akt pathway and downstream mitochondria-mediated and caspase-dependent signalling pathways. 

### 3.2. The Relationship between Antioxidant, Neuroprotective Activity, and Chemical Structure of Stilbenoids

Oxidative stress induced by ROS overproduction is an important mechanism by which Aβ exerts its neurotoxicity. Therefore, the neuroprotective activity of stilbenoids against Aβ is determined by their own ROS-scavenging activity to some extent.

THS, RES, and PIC significantly decreased the levels of DPPH• and •OH (Figure 2) and intracellular ROS (Figure 3A), demonstrating antioxidant activities. In addition, the antioxidative effects indicated that the antioxidant activity of the stilbenoids increased with the number of hydroxyl substituents to a certain extent. This result was consistent with previous observation that hydroxyl groups on RES enhance free radical scavenging activity towards superoxide (O^2^•^−^) and DPPH• radicals for resveratrol analogues [21].

Regarding neuroprotective activity, cell viability increased with the presence of the three stilbenoids in Aβ-induced primary neurons. THS and RES increased cell viability to a similar extent, whereas both were weaker than PIC (Figure 3B). With respect to the signalling pathway, PIC showed the best neuroprotective activity in promoting Akt phosphorylation, stimulating Bcl-2/Bax expression, and inhibiting PARP cleavage (Figure 4 and Figure 5). Based on the results, we conclude that the neuroprotective activity of stilbenoids is associated with the number as well as the position of hydroxyl substituents. Previous studies have indicated that polyhydroxylated stilbenoids with a 3,4-dihydroxyl group (*ortho*-dihydroxy structure) or 3,4,5-trihydroxyl group (*ortho*-trihydroxy structure) have higher antioxidant capacities and exert more pronounced antiproliferative activities than RES [6,21]. The result pertaining to the structure–activity relationship of another class of polyphenolic compounds (flavonoids) showed a similar pattern in which the *ortho*-dihydroxy structure in ring B of the flavonoids led to more effective antioxidant capacities [40]. We hypothesized that PIC showed the highest antioxidant and neuroprotective ability, most likely the result of the 3,4-dihydroxyl (*ortho*-dihydroxy) structure on a benzene ring. The *ortho*-dihydroxy group in PIC can scavenge a ROS free radical to form a more stable oxidation intermediate, *ortho*-semiquinone, as a result of the stronger intramolecular hydrogen bonding interaction, as indicated by both theoretical calculations [41] and experiments [42]. Moreover, *ortho*-semiquinones will be deprotonated to semiquinone anions, which will scavenge an additional ROS free radical to form the final product *ortho*-quinone [43]. As a result, PIC is more potent to relieve the oxidative stress induced by ROS and showed a greater neuroprotective ability.

In contrast, RES with a *meta*-dihydroxy group cannot form a stabilized semiquinone structure but forms only neutral phenoxyl radicals, which would decay rapidly by disproportionation (reaction (1)) or dimerization (reaction (2)) [21]. Therefore, the *meta*-dihydroxy group on RES had low activity in scavenging free radicals, which is consistent with a previous observation that stilbenoid with only a 3,5-dihydroxyl group (*meta*-dihydroxy group) was resistant to oxidation [44]. In addition, theoretical calculations had proven that the 4’-hydroxy radical of resveratrol is the most stable form. Therefore, the 4’-hydroxy group is much more easily oxidized than other hydroxyl groups [45], which might be the reason why RES with two more hydroxy groups possessed the same or similar neuroprotective ability compared with THS.

HO-stilbene-O•+ HO-stilbene-O•→ HO-stilbene-OH + O=stilbene=O(1)

HO-stilbene-O•+ HO-stilbene-O•→ Dimers(2)

In conclusion, the antioxidant and neuroprotective activity of stilbenoids is related to the number of hydroxyl substituents to a certain extent. Moreover, the position of hydroxyl groups is significant; stilbenoids with an *ortho*-dihydroxy structure possess a greater neuroprotective activity, whereas the *meta*-dihydroxy structure has no impact on neuroprotective activity.

## 4. Materials and Methods 

### 4.1. Reagents and Chemicals 

THS (≥99%), RES (≥99%), and PIC (≥99%) were obtained from J&K Scientific, Inc. (Beijing, China). Aβ_25-35_ (≥99%) was purchased from Chinese Peptide, Inc. (Beijing, China). Neurobasal, B27, 0.25% trypsin, sodium pyruvate, and 2-mercaptoethanol were purchased from Gibco (Grand Island, NY, USA). Fetal bovine serum was purchased from Hyclone Laboratory, Inc. (Logan, UT, USA). Furthermore, 3-[4,5-Dimethylthiazol-2-yl]-2,5-diphenyl tetrazolium bromide (MTT) was obtained from AMRESCO, Inc. (Solon, OH, USA). Also, 2′,7′-Dichlorofluorescin diacetate (DCFH-DA) was obtained from Beijing Solarbio Science & Technology Co., Ltd. (Beijing, China). The following rabbit antibodies were purchased from Cell Signalling Technology, Inc. (Boston, MA, USA): phosphor-Bad (Ser-136), phosphor-Akt (Ser-473), Akt, Bax, Bcl-2, caspase-8, cleaved caspase-3, cleaved caspase-9, and cleaved PARP. Other analytical grade regents were obtained from Beijing Chemical Reagent, Inc. (Beijing, China).

### 4.2. Cell Culture and Peptide Preparation

The study was preformed according to the guidelines approved by China Agricultural University regarding the care and use of animals for experimentation (approval letter 20135001-3, dated 3 December 2016). Aβ_25-35_ peptide was dissolved in PBS solution at 1 mM and incubated for 14 days at 37 °C for aggregation [46]. The solution was stored at −20 °C until use. Primary cerebral cortex neurons were prepared from Sprague–Dawley rats embryos of day 16–18 (E16–18) as described previously [47]. Briefly, embryos were removed, then cerebral cortex neurons were dissected out and triturated in serum-free culture medium after meninges were removed. Tissue was incubated in 0.25% trypsin for 25 min at 37 °C and then mechanically triturated. The cells were collected and clarified by centrifugation (600 g, 5 min). Cells were resuspended in Neurobasal medium supplemented with 2% B27, 0.5 mM L-glutamine, 25 μM 2-mercaptoethanol, and 100 U/mL penicillin/streptomycin. Cells were placed on 96-well plates or 6-well plates with poly-d-lysine coated for different assays. Cells were stored at 37 °C in a humidified atmosphere of air and 5% CO_2_. After three days, neurons were randomly divided into a control group, model group, and different-dose stilbenoids group. The control group included cells that grew normally in the absence of any stimulation. The model group was treated with Aβ_25-35_ for 24 h. The different-dose stilbenoids group was treated with different concentrations of stilbenoids for 1 h and then replaced with Aβ_25-35_ for 24 h.

### 4.3. In Vitro Antioxidant Activity

Antioxidant activity assay of scavenging free radicals was performed by the same procedure as described previously [48]. Electron paramagnetic resonance (EPR) spectroscopy measurements were performed on DPPH• and •OH stabilized radical models. The EPR experiments were run on a FA-300 spectrometer (JEOL, Japan) with the following typical settings: 1. DPPH• model: centre field 336 mT, sweep width 7.5 mT, modulation width 0.35 mT, and amplitude 50. Ten microlitres of 2 μmol/L of each stilbenoid was added individually to 10 μL of a freshly made 2 mmol/L DPPH• solution; 2. •OH model: centre field 336 mT, sweep width 7.5 mT, modulation width 0.35 mT, and amplitude 200. Five microlitres of 2 μmol/L of each stilbenoid was added individually to a solution consisting of 5 μL of ferrous sulfate, 5 μL of DMPO, and 5 μL of fresh 1% H_2_O_2_. The free radical scavenging rate for the EPR spectra was calculated according to the decrease in peak intensity. Inhibition (%) = (I_0_ − I_S_) / I_0_ × 100, where I_0_ is the intensity without the extract and I_S_ is the intensity of the stilbenoids.

### 4.4. Intracellular Reactive Oxygen Species (ROS) Measurement

The level of intracellular ROS was detected with the DCFH-DA assay as described previously [49]. DCFH-DA reacts with ROS in cells and is converted to a fluorescent product, DCF, which can be detected by flow cytometry. Briefly, neurons were seeded into 6-well plates at 1.0 × 10^6^ cells/well. After drug treatment, the cells were incubated with 5 μM DCFH-DA for 30 min at 37 °C, followed by three washes with PBS. The fluorescence of DCF was detected by flow cytometry (BD, San Jose, CA, USA) at Ex = 488 nm and Em = 605 nm. ROS production was expressed as the fold change relative to untreated controls.

### 4.5. Cell Viability 

Cell viability assay was performed by using the MTT assay. Briefly, 4 × 10^5^ cells/well were plated in 96-well plates for 24 h. The stilbenoid groups were treated with different concentrations of stilbenoids for 1 h. After removed the medium, the model group and different-dose stilbenoids groups were treated with 50 μM Aβ_25-35_ for 24 h. Then, the medium was replaced with 100 μL/well of MTT medium (MTT final concentration 0.5 mg/mL). The plates were incubated at 37 °C for 4 h, allowing viable cells to reduce the yellow tetrazolium salt to dark blue formazan crystals. After incubation, MTT solubilization solution (10% Triton X-100, 50% isopropanol, and 40% double distilled water) was added to the wells to dissolve formazan crystals at 37 °C for 6 h. Finally, the absorbance of each individual well was determined at 570 nm with a microplate reader (Thermo multiskan Mk3, Thermo Fisher Scientific Inc., Waltham, MA, USA). Cell viability was expressed as a percentage of the untreated controls.

### 4.6. Western Blotting 

For Western blotting analysis, primary cortical neurons were seeded into 6-well plates at 1.0 × 10^6^ cells/well. After treatment, the cells were washed twice with PBS, harvested, and centrifuged at 600 g for 5 min. The cells were collected with 100 μL of lysis buffer on ice for 30 min, followed by ultrasonic degradation 10 times at 30 W with an Ultrasonic Processor (Scientz, Ningbo, China). The supernatant was obtained by centrifugation at 12,000× *g* for 30 min. The protein concentrations were measured with the BCA protein assay kit (Beijing Solarbio Science & Technology Co., Ltd., Beijing, China). Equal protein solutions were mixed with 4× loading buffer and heated to 100 °C for 6 min. Proteins were separated on an 8% or 10% SDS-polyacrylamide gel, then transferred onto a PVDF membrane for 100 min at 200 mA in a wet transfer system (Bio-Rad, Hercules, CA, USA). The membrane was blocked with 5% non-fat milk and 0.1% Tween-20 in TBST buffer for 1 h at room temperature. Then, the membrane was incubated overnight with primary antibodies, including p-Bad, p-Akt, Akt, Bax, Bcl-2, caspase-8 c-caspase-3, c-caspase-9, c-PARP, and β-actin diluted 1:1000 at 4 °C. After five washes with TBST containing 0.1% Tween-20 for 8 min each, the membrane was incubated with HRP-conjugated secondary antibodies diluted 1:5000 for 1 h at room temperature. After another five washes with TBST for 8 min, the membrane was visualized using Western blotting luminal reagents. The results were analyzed with ImageJ software (http://rsbweb.nih.gov/ij/download.html). The expression level was corrected to β-actin.

### 4.7. Molecular Docking

Akt protein was taken from the X-ray structure at 1.49 Å resolution in the Protein Data Bank (chain A of PDB entry 4GV1). PIC was obtained from X-ray structure at 2.3 Å structure (ligand of PDB entry 4HD8). RES and THS were taken from X-ray structure at 1.5 Å structure (ligand of PDB entry 1SG0). Three stilbenoids were then docked against Akt protein using AutoDock 4 (http://autodock.scripps.edu). A grid box with three dimensions at 100 × 125 × 70 Å and a grid spacing of 0.375 Å was set up by centering on x = −20.370, y = 2.030, and z = −5.000. The docking site was set on the COOH-terminal region located in the grid center; 100 runs were performed. The strength of the combination affinity of the ligand and receptor was calculated according to the binding energy. The docking poses were displayed and analyzed by PyMOL viewer (http://www.pymol.org/). The hydrogen bonds and hydrophobic interactions were analysed using LigPlot+ analysis [50].

### 4.8. Statistical Analysis

All data were expressed as mean ± SD values (n = 3). One-way analysis of variance (ANOVA) was used to compare multiple groups using SPSS 19.0 (SPSS Inc., Chicago, IL, USA). Different letters (a, b, c, etc.) indicate significant differences by Duncan’s multiple range test at *p* < 0.05. 

## 5. Conclusions

The present study demonstrates that THS, RES, and PIC possess antioxidant and neuroprotective activity against Aβ_25-35_-induced neurotoxicity in rat primary cortex neurons. In particular, THS, RES, and PIC were able to scavenge DPPH• or •OH free radicals and inhibited intracellular ROS accumulation in neurons. All three tested stilbenoids exhibited remarkable neuroprotective activity against Aβ_25-35_-induced neurotoxicity via the PI3K/Akt signalling pathway and downstream mitochondria-mediated and caspase-dependent signalling pathways. Molecular docking results indicated that stilbenoids could form hydrogen bond interactions with the COOH-terminal region of Akt. The antioxidant and neuroprotective activities of the stilbenoids are correlated with the number and position of hydroxyl groups. The stilbenoids bearing ortho-dihydroxyl functionality exhibited significantly higher antioxidative and neuroprotective activity.

## Figures and Tables

**Table 1:** Binding Energy of *trans*-4-hydroxystilbene (THS), resveratrol (RES), and piceatannol (PIC) and Akt protein docking systems.

Stilbenoids	Binding Energy (Kcal/mol)	*K_i_* (μM)	Final Intermolecular Energy (Kcal/mol)	VDW + H Bond + Desolvation Energy (Kcal/mol)	H Bonds
*trans*-4-hydroxystilbene	−6.31 ± 0.33	23.78	−7.2	−7.1	THS:O1H1-GLU432:O
					ASN231:ND2-THS:O1
resveratrol	−6.69 ± 0.68	12.54	−8.18	−7.82	RES:O1-LEU213:O
					RES:O2-TYR229:O
					LYS284:NZ-RES:O2
					TYR175:HN-RES:O3
piceatannol	−6.73 ± 0.51	11.75	−8.52	−8.28	PIC:HAD-ILE447:O
					PIC:H1-ILE447:O
					PIC:H2-PRO451:O
					PIC:H3-PHE150:O
					PIC:H3-MET147:O
